# Beyond the relationship between mitochondria and mobility in aging

**DOI:** 10.18632/aging.204649

**Published:** 2023-06-12

**Authors:** Qu Tian, Luigi Ferrucci

**Affiliations:** 1 Longitudinal Studies Section, Translational Gerontology Branch, National Institute on Aging, Baltimore, Maryland 21224, US

**Keywords:** mobility, mitochondria

Cognitive decline and physical function deterioration are common but critical manifestations of aging, both affecting independence and quality of life in older age. Physical deterioration, such as slow gait or mobility decline, is a strong predictor of multiple health outcomes, including cognitive impairment, dementia, and mortality. It is recognized that mobility decline stems from deficits in multiple systems, such as musculoskeletal, cardiopulmonary, and central nervous systems. Understanding the biological underpinnings of mobility decline is key to designing future prevention and intervention strategies to prevent mobility decline, improve quality of life, and extend health span. Mitochondrial dysfunction, one of the aging hallmarks, may target many aspects of the aging process which contribute to subsequent mobility decline. Assessing mitochondrial function in large epidemiological studies has its challenges, as mitochondria exist in various tissues and circulation and because the physiological functions of mitochondria extend beyond the production of energy in the form of adenosine triphosphate. One powerful *in vivo* assessment in the skeletal muscle is to measure oxidative capacity by capturing the post-exercise recovery rate of phosphocreatine using MR spectroscopy. This metric has been associated with permeabilized muscle fibers' respiration [1].

In our recent study using data from the Baltimore Longitudinal Study of Aging, we examined the relationship between skeletal muscle mitochondrial function and mobility decline in a sample of initially well-functioning older adults who had an initial gait speed of equal to or greater than 1.0 meter/second. Across multiple mobility measures, we found consistent associations between higher skeletal muscle mitochondrial function and a slower decline in mobility performance [2]. This relationship was only slightly attenuated after accounting for longitudinal assessments of muscle strength and mass. These findings suggest that mitochondria contribute to mobility decline even in an early stage of being well-functioning, and declining muscle function may play a role in this relationship. This study extends the prior cross-sectional examination of skeletal muscle mitochondrial function and mobility [3], highlights the importance of muscle function, and provides new insights into biological causes of mobility decline.

In addition to the important role of muscle function, the connection between mitochondrial dysfunction and mobility decline may also involve the brain. Both muscle and brain are energy-demanding organs and are related to mobility decline [4]. During the aging process, the involvement of mitochondria highly depends on oxidative metabolism and therefore is tissue specific. Mitochondria are highly involved in the brain, skeletal muscle, and heart demonstrated by studies of gene expression profiling [5]. As neurons and myocytes are post-mitotic, damaged organelles cannot be diluted by cellular division. Instead, both neurons and myocytes rely on mitochondrial quality control and mitophagy to maintain mitochondrial homeostasis and preserve mitochondrial function. Thus, a subtle disruption in mitochondrial quality control pathways may lead to mitochondrial dysfunction, altered energy metabolism, and ultimately neuronal and myocyte damage. The essential role of mitochondrial dysfunction in muscle function, such as sarcopenia, has been studied in both animal and human studies, but data on the relationship between mitochondrial function and brain function are sparse, especially in humans. Recently, a meta-analysis conducted in several aging cohorts across the US have shown that mitochondrial DNA copy number, a blood-based marker from Whole Genome Sequencing data, is associated with cognitive function [6].

Epidemiological evidence informs and connects hypotheses for basic biology and intervention studies in humans. More epidemiological studies are needed to extend the understanding of the role of mitochondrial dysfunction in mobility decline, such as other metrics of mitochondrial function across various tissues and circulation, and shared biomarker signatures of mitochondrial function and mobility decline. Studies from our group found that longitudinal change in skeletal muscle mitochondrial function was associated with simultaneous changes in specific lipid metabolites of lysophosphatidylcholines and these specific metabolites were also associated with mobility performance [7, 8]. Lysophosphatidylcholines are precursors of cardiolipin, a phospholipid and building blocks of the inner mitochondrial membrane. Targeting these mitochondrial-related biomarkers may preserve mitochondrial function and mobility function. Human studies have shown improved mitochondrial function through pharmacological and non-pharmacological (e.g. physical exercise) approaches [4]. Whether these approaches improve mobility function and prevent mobility decline through improved mitochondrial function warrants further investigation.
